# The E3 ligase TRIM22 restricts SARS-CoV-2 replication by promoting proteasomal degradation of NSP8

**DOI:** 10.1128/mbio.02320-23

**Published:** 2024-01-26

**Authors:** Lujie Fan, Yuzheng Zhou, Xiafei Wei, Wei Feng, Huimin Guo, Yunfei Li, Xiang Gao, Jian Zhou, Yanling Wen, Yezi Wu, Xiaotong Shen, Lei Liu, Gang Xu, Zheng Zhang

**Affiliations:** 1 Guangzhou Laboratory, Guangzhou Medical University, Guangzhou, China; 2 Institute for Hepatology, National Clinical Research Center for Infectious Disease, Shenzhen Third People’s Hospital, The Second Affiliated Hospital, School of Medicine, Southern University of Science and Technology, Shenzhen, Guangdong Province, China; 3 Department of Microbiology, School of Basic Medical Sciences, Anhui Medical University, Hefei, China; 4 Guangdong Key laboratory for Anti-infection Drug Quality Evaluation, Shenzhen, Guangdong, China; 5 Shenzhen Research Center for Communicable Disease Diagnosis, Treatment of Chinese Academy of Medical Science, Shenzhen, Guangdong, China; Stony Brook University, Stony Brook, New York, USA

**Keywords:** SARS-CoV-2, NSP8, TRIM22, ubiquitination, protein degradation

## Abstract

Replication of severe acute respiratory syndrome coronavirus 2 (SARS-CoV-2) genome is mediated by a complex of non-structural proteins (NSPs), of which NSP7 and NSP8 serve as subunits and play a key role in promoting the activity of RNA-dependent RNA polymerase (RdRp) of NSP12. However, the stability of subunits of the RdRp complex has rarely been reported. Here, we found that NSP8 was degraded by the proteasome in host cells, and identified tripartite motif containing 22 (TRIM22) as its E3 ligase. The interferon (IFN) signaling pathway was activated upon viral invasion into host cells, and TRIM22 expression increased. TRIM22 interacted with NSP8 and ubiquitinated it at Lys97 via K48-type ubiquitination. TRIM22 overexpression significantly reduced viral RNA and protein levels. Knockdown of TRIM22 enhanced viral replication. This study provides a new explanation for treating patients suffering from SARS-CoV-2 with IFNs and new possibilities for drug development targeting the interaction between NSP8 and TRIM22.

**IMPORTANCE:**

Non-structural proteins (NSPs) play a crucial role in the replication of severe acute respiratory syndrome coronavirus 2, facilitating virus amplification and propagation. In this study, we conducted a comprehensive investigation into the stability of all subunits comprising the RNA-dependent RNA polymerase complex. Notably, our results reveal for the first time that NSP8 is a relatively unstable protein, which is found to be readily recognized and degraded by the proteasome. This degradation process is mediated by the host E3 ligase tripartite motif containing 22 (TRIM22), which is also a member of the interferon stimulated gene (ISG) family. Our study elucidates a novel mechanism of antiviral effect of TRIM22, which utilizes its own E3 ubiquitin ligase activity to hinder viral replication by inducing ubiquitination and subsequent degradation of NSP8. These findings provide new ideas for the development of novel therapeutic strategies. In addition, the conserved property of NSP8 raises the possibility of developing broad antiviral drugs targeting the TRIM22-NSP8 interaction.

## INTRODUCTION

Since the emergence of coronavirus disease 2019 (COVID-19) pandemic caused by severe acute respiratory syndrome coronavirus 2 (SARS-CoV-2), extensive studies have been conducted to understand the structural and functional characteristics of this novel coronavirus (1, 2). SARS-CoV-2 is an enveloped, positive-sense, single-stranded RNA virus consisting of 29 proteins, including structural proteins (spike, membrane, envelope, and nucleocapsid proteins), non-structural proteins (NSP1-16), and multiple accessory proteins (1, 3). The spike protein binds to angiotensin-converting enzyme 2 receptor via the receptor-binding domain and enters the host cell via membrane fusion mediated by the S2 domain (4, 5). RNA-dependent RNA polymerase (RdRp) is key to the replication and transcription of SARS-CoV-2 and is composed of NSP12, NSP7, and NSP8 (1, 6–8). NSP12 catalyzes viral RNA synthesis, while NSP8, in conjunction with the cofactor NSP7, activates and sustains viral RNA production. NSP8 plays a critical role in recognizing viral RNA and forming complexes, thereby facilitating the transcription and replication of viral RNA. The complex formed by NSP12, NSP7, NSP8, and the RNA duplexes is referred to as the central replication-transcription complex (RTC) and serves as a marker of RdRp activity (6, 8). In addition, NSP8 possesses immunomodulatory properties. It can inhibit the production of type I and type III interferons (IFNs) by targeting key signaling molecules such as RIG-I/MDA5, TRIF, and STING (9). Furthermore, NSP8 can damage the mitochondria and induce incomplete mitophagy (10). These findings highlight the multifunctionality of NSP8 and its potential role in modulating host immune response and cellular processes during SARS-CoV-2 replication. Understanding the biological characteristics of NSP8 is of paramount importance for developing effective antiviral strategies against SARS-CoV-2. However, our current knowledge on NSP8 remains limited, necessitating further studies to fully elucidate its role and as a potential therapeutic target.

Tripartite motif containing (TRIM) 22, also known as Staf-50, is a member of the TRIM family of proteins (11). The TRIM family comprises highly conserved proteins that possess a RING finger, B-box, and coiled-coil structural domain (12). TRIM proteins have diverse cellular functions, including inhibition of viral replication and regulation of the innate immune response (13–15). Recently, the TRIM family has been recognized as IFN-inducible proteins involved in cell proliferation and antiviral defense (16). TRIM22 acts as a natural antiviral effector against human immunodeficiency virus 1 (17), hepatitis B virus (18), and influenza (17). Its antiviral function depends on its ability to ubiquitinate target proteins and varies across different viruses. For instance, TRIM22 targets the influenza nucleoprotein for degradation (17), promotes non-structural 5A degradation to inhibit hepatitis C virus (12), targets Janus kinase-signal transducers and activators of transcription 1/2 signaling to inhibit the replication of respiratory syncytial virus (19), and inhibits core promoter activity of hepatitis B virus (18). Moreover, TRIM22 inhibits endometrial cancer progression and improves prognosis by targeting the nucleotide binding oligomerization domain containing 2 (NOD2)/nuclear factor κB (NF-κB) signaling pathway (20). However, the role of TRIM22 in SARS-CoV-2 replication is unknown.

SARS-CoV-2 invasion disrupts host cell homeostasis, and protein ubiquitination can act as a signaling molecule to degrade them via the proteasome, as well as to alter protein cellular localization and affecting their function (21). SARS-CoV-2 proteins are also regulated by ubiquitination in the host. TRIM21 promotes the ubiquitination of SARS-CoV-2 coat proteins to regulate innate immunity (22); ubiquitination of NSP6 and ORF7a promotes the activation of NF-κB (23); and Ring-finger protein 5 (RNF5) restricts SARS-CoV-2 replication by degrading its envelope protein (24). However, there are few reports on the ubiquitination of RdRp complexes. In this study, we focus on the stability of the SARS-CoV-2 RdRp complex for in-depth studies, aiming to find antiviral molecules that directly target RdRp, thus providing fundamental support for the development of broad antiviral drugs.

## RESULTS

### NSP8 is degraded by the ubiquitin-proteasome pathway

**Fig 1:**
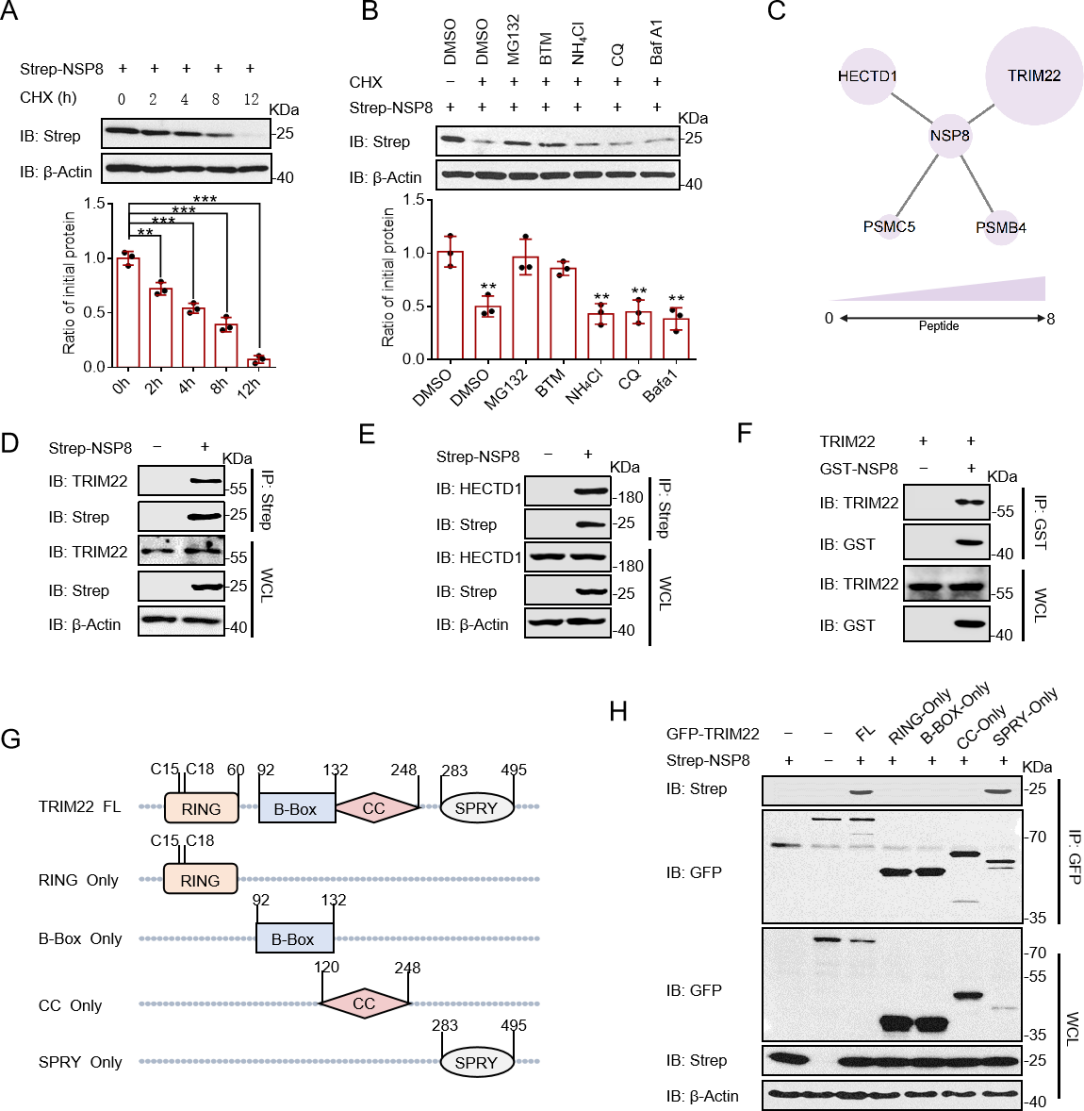
NSP8 is degraded by the ubiquitin proteasome pathway and interacts with TRIM22. (**A**) HEK-293T cells in a 6 cm dish were transfected with the indicated Strep-tagged plasmids. Twelve hours later, cells were split evenly into wells of a 24-well plate and treated with CHX (25 µg/mL) when 100% confluence was reached. Cells were collected at the indicated times and the levels of NSP8 were detected with anti-Strep antibody. (**B**) HEK-293T cells transfected with Strep-NSP8 plasmid were treated with dimethyl sulfoxide (DMSO), CHX (25 µg/mL), MG132 (20 µM), BTM (10 µM), CQ (20 µM), and NH_4_Cl (10 mM) for 8 h and then collected. The protein level of NSP8 was detected by Western blotting. Quantification of NSP8 protein levels relative to β-actin is shown. (**C**) Proteome overlays interacting with NSP8 were identified by mass spectrometry, represented in circles of different sizes according to the number of peptide segments. (**D**) HEK-293T cells transfected with Strep-NSP8 and vector were lysed with NP-40. The whole-cell lysates were subjected to pulldown with anti-Strep beads and Western blotting to detect TRIM22. (**E**) Glutathione S-transferase (GST)-NSP8 was purified from *Escherichia coli*. The beads binding the target proteins were incubated with the whole-cell lysates from HEK-293T cells, and the pulldown proteins were detected with anti-GST antibody by immunoblotting. (**F**) Schematics of different domains of TRIM22. (**G**) HEK-293T cells transfected with Strep-NSP8 and the different domains of TRIM22 with green fluorescent protein (GFP)-Tag expression plasmid. The whole-cell lysates were subjected to pulldown with anti-GFP antibody. (**H**) The described plasmids were transfected into HEK-293T cells, and Strep-NSP8 and TRIM22 were detected by immunoblotting and analyzed in grayscale. Results expressed as mean + SD (*n* = 3 independent experiments). * indicates *P* < 0.05, ** indicates *P* < 0.01, and *** indicates *P* < 0.001, and statistics used Student’s *t*-test.

The RdRp complex of SARS-CoV-2 is mainly composed of three non-structural proteins including NSP7, NSP8, and NSP12. We examined the stabilities of NSP7, NSP8, and NSP12 in cycloheximide (CHX)-treated cells and found that NSP8 was more unstable than NSP7 and NSP12 (Fig. 1A; Fig. S1A and B). To investigate the pathway involved in NSP8 degradation, we treated cells with proteasome inhibitors (MG132 and bortezomib [BTM]) or lysosomal inhibitors (NH_4_Cl, chloroquine [CQ], and bafilomycin A1). The results showed that proteasome inhibitors, but not lysosomal inhibitors, mostly inhibited NSP8 degradation (Fig. 1B). Therefore, NSP8 was mainly degraded via the ubiquitin-proteasome pathway. MG132 significantly inhibited NSP8 degradation at 0, 2, 4, and 8 h after CHX treatment (Fig. S1C).

TRIM7 targets NSP8 for proteasomal degradation via K48-linked polyubiquitination of NSP8 (25). However, whether TRIM7 is the sole E3 ligase of NSP8 is unclear. To address this issue, we first validated the effect of TRIM7 on NSP8 expression. Surprisingly, NSP8 levels decreased after CHX treatment, even in the low level of TRIM7. However, the degradation rate of NSP8 was lower than that of the conventional level (Fig. S1D). MG132 and BTM significantly increased NSP8 levels (Fig. S1E and F). These results indicate that NSP8 is an unstable protein that can be rapidly degraded via the ubiquitin-proteasome pathway and undergoes substantial ubiquitin-proteasome-mediated degradation despite TRIM7 knockdown, suggesting that other E3 ligases might be involved in regulating NSP8.

To identify other E3 ligases targeting NSP8, we expressed NSP8 with a Strep-tag in HEK-293T cells and performed protein profiling using anti-Strep-enriched NSP8 (Fig. S1G). The results indicated TRIM22 and HECT domain E3 ubiquitin protein ligase 1 (HECTD1) as E3 ligases, with TRIM22 showing the highest abundance. Additionally, some proteasome subunits (proteasome 26S subunit, ATPase [PSMC] 4 and 5) were detected (Fig. 1C; Table S1). Immunoprecipitation (IP) assay showed a strong interaction between NSP8 and endogenous TRIM22 and HECTD1 (Fig. 1D and E). Similarly, IP results showed that NSP8 interacted with exogenous TRIM22 (Fig. S1H and I). To examine whether NSP8 and TRIM22 directly interact, purified glutathione S-transferase (GST)-NSP8 and TRIM22 were incubated, and an IP assay was performed using anti-GST agarose beads. The results showed that TRIM22 directly interacted with NSP8 (Fig. 1F; Fig. S1J). TRIM22 contains four structural domains, including RING, B-box, coiled-coil (CC), and SPRY^5^. To identify which structural domain of TRIM22 interacts with NSP8, a series of TRIM22-truncated plasmids were constructed (Fig. 1G). Our results indicated that the SPRY domain was responsible for this interaction (Fig. 1H). Finally, we used a protein interaction site prediction website to predict the major sites for hydrogen bond formation between NSP8 and TRIM22 and found that NSP8 and TRIM22 might form hydrogen bonds at these sites (L122-Y398, T123-Q413/N414, T124-Q413, S151/R190-V392, R190/S151-V392, N192-N393), thus causing the interaction (Fig. S1L). Subsequently, point-mutated NSP8 was constructed and verified by co-IP. The results showed that the T124, R190, and R192 sites of NSP8 were mutated and their binding to TRIM22 was reduced, suggesting that these three amino acid sites may play a role in NSP8-TRIM22 complex (Fig. S1M). However, the results also showed that single mutation could still interact with NSP8-TRIM22. We hope to provide some reference value for identifying the interaction sites between NSP8 and TRIM22. These results suggest that TRIM22 and HECTD1 interact with NSP8.

### TRIM22 promotes ubiquitination-mediated proteasomal degradation of NSP8

**Fig 2:**
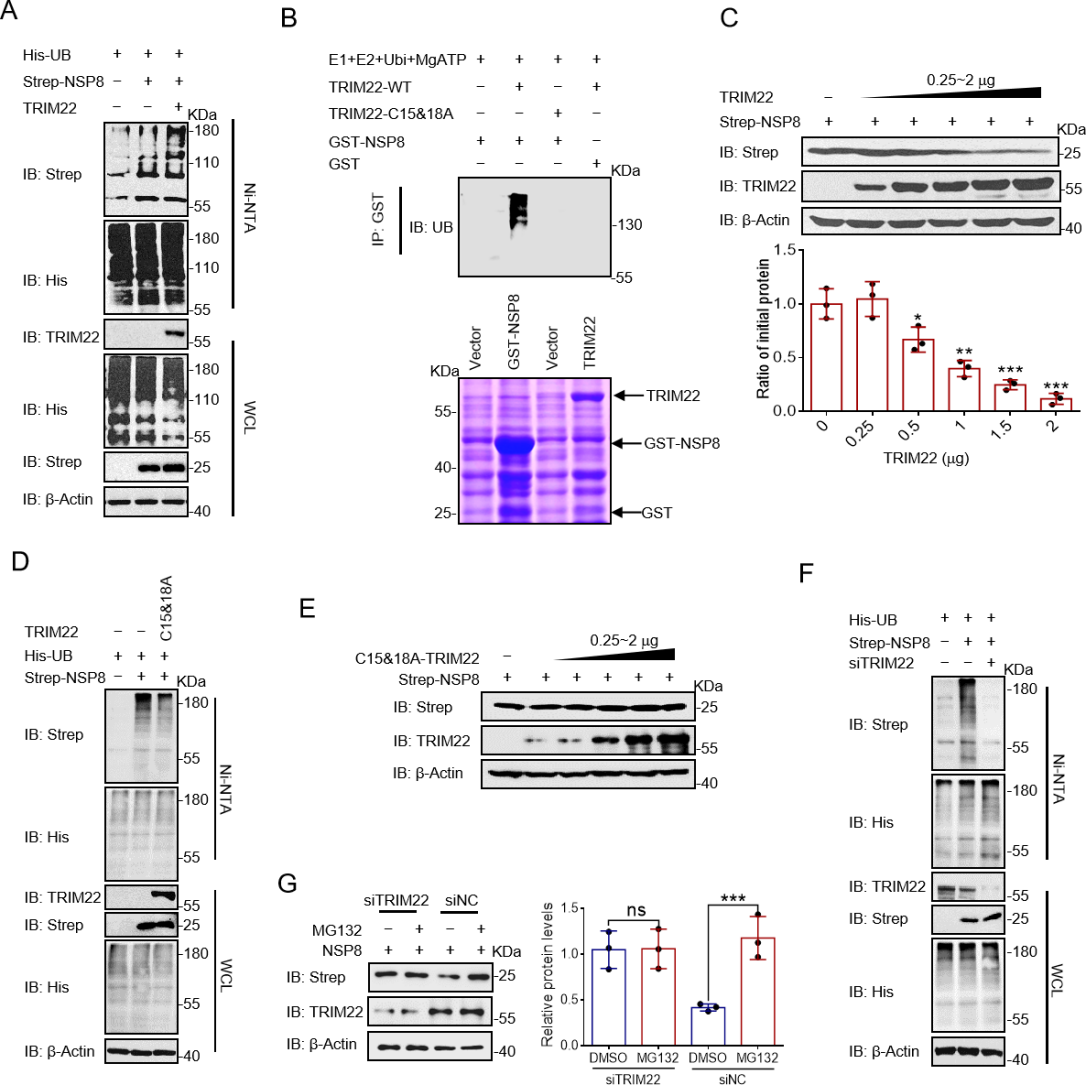
TRIM22 promotes ubiquitin proteasomal degradation of NSP8. (**A, D, F**) The plasmids described were transfected into HEK-293T cells and the ubiquitination of NSP8. Cells were treated with MG132 (20 µM) for 8 h prior to collection. The whole-cell lysates were subjected to pulldown with Ni-NTA beads and Western blotting to detect the polyubiquitination chain of NSP8. (**B**) Proteins were co-complemented in groups as described, and after enrichment of GST-NSP8 proteins with anti-GST beads, they were detected with anti-Ub antibody. (**C**) The NSP8 ubiquitination linkage was analyzed in HEK-293T cells transfected with NSP8 and the indicated ubiquitin-wild type (WT) only retained Lys-29, Lys-33, Lys-48, and Lys-63 plasmids. Cells were treated with MG132 (20 µM) or not for 8 h prior to collection. The whole-cell lysates were subjected to pulldown with Ni-NTA beads and Western blotting to detect the polyubiquitination chain of NSP8. (**E and G**) The plasmids described were transfected into HEK-293T cells, and the NSP8 protein was detected by Western blotting with anti-Strep antibody, quantitative display of NSP8 protein levels relative to β-actin. Results expressed as mean + SD (*n* = 3 independent experiments). * indicates *P* < 0.05, ** indicates *P* < 0.01, and *** indicates *P* < 0.001, and statistics used Student’s *t*-test.

To investigate the role of TRIM22 and HECTD1 as E3 ubiquitin ligases for NSP8, their ability to modify NSP8 ubiquitination was examined. The results showed that TRIM22 overexpression markedly increased the ubiquitination of NSP8, whereas HECTD1 overexpression did not (Fig. 2A; Fig. S2A). To further demonstrate that TRIM22 can directly promote NSP8 to undergo ubiquitination, an *in vitro* ubiquitination assay was performed, and the results showed that TRIM22 promoted the ubiquitination of NSP8 (Fig. 2B). Overexpression of TRIM22 decreased NSP8 level (Fig. 2C; Fig. S2B). Cysteine residues at positions 15 and 18 are the catalytically active sites of TRIM22 (26), and the C15 and 18A mutants of TRIM22 could not catalyze the ubiquitination of NSP8 (Fig. 2D; Fig. S2C and D). Moreover, TRIM22 with C15 and 18A mutations did not promote NSP8 degradation (Fig. 2E; Fig. S2E). TRIM22 knockdown significantly reduced the ubiquitination of NSP8 (Fig. 2F), and MG132 did not rescue NSP8 levels (Fig. 2G).

Eight types of ubiquitinations (M1, K6, K11, K27, K29, K33, K48, and K63) have been previously reported (27), each with different functions. The K48 type promotes substrate degradation by the proteasome (28). To investigate the type of polyubiquitination of NSP8 catalyzed by TRIM22, different ubiquitin plasmids containing TRIM22 and NSP8 were co-transfected into HEK-293T cells for His pulldown. The results showed that only total ubiquitin and K48 ubiquitin chains were detected (Fig. S2F). This suggests that TRIM22 catalyzes K48 polyubiquitination of NSP8, thereby promoting its proteasomal degradation.

### TRIM22 induces NSP8 degradation by enhancing ubiquitination at K97 site

**Fig 3:**
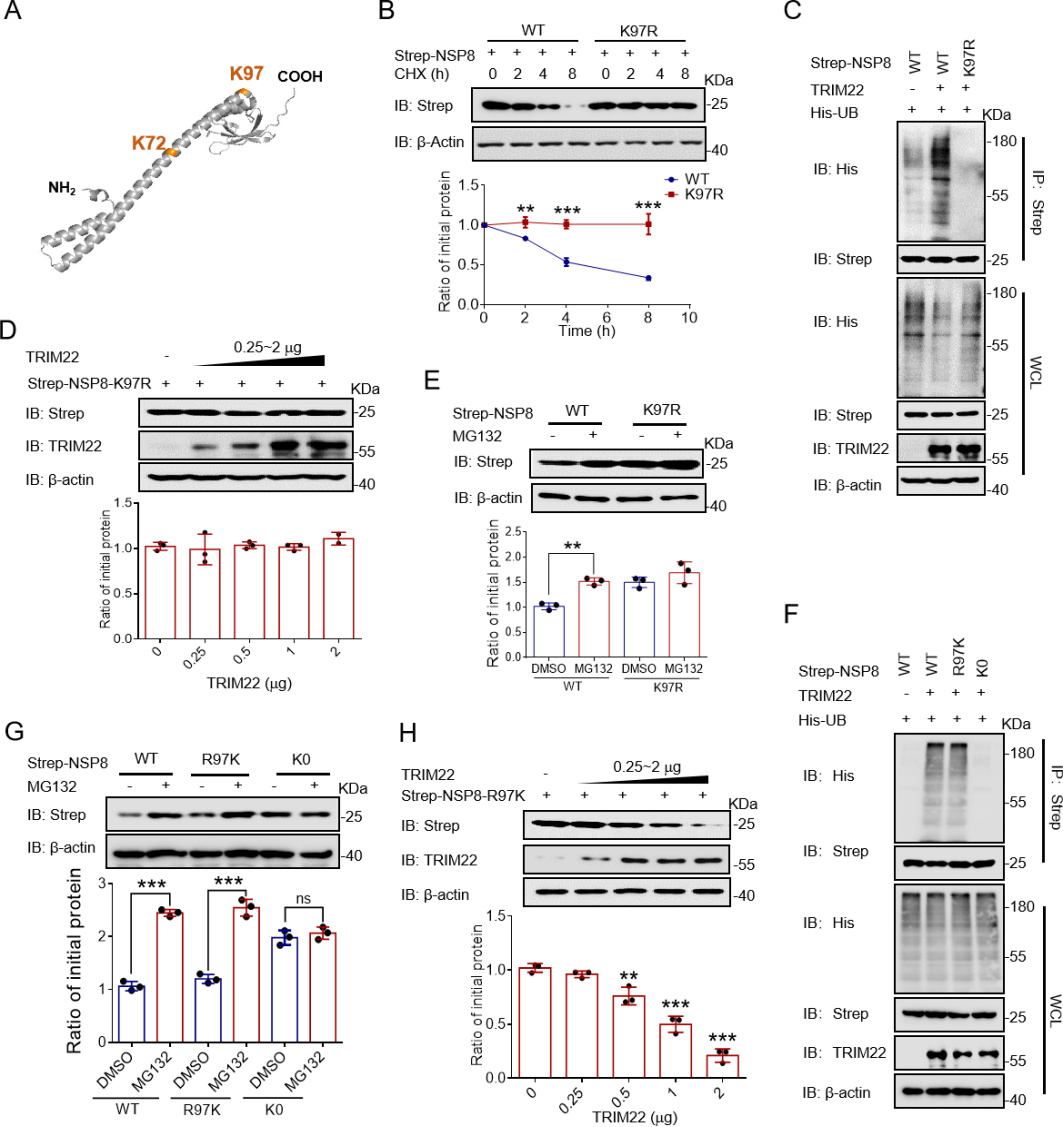
TRIM22 induces the degradation of NSP8 by enhancing its ubiquitination on K97. (**A**) Schematic representation of the ubiquitination site of the NSP8 protein. (**B**) NSP8-WT or NSP8-K97R plasmids were transfected into HEK-293T cells, treated with CHX (25 µg/mL), and then detected by Western blotting with the indicated antibodies. (**C and F**) The plasmids described were transfected into HEK-293T cells and the ubiquitination of NSP8. Cells were treated with MG132 (20 µM) for 8 h prior to collection. The whole-cell lysates were subjected to pulldown with anti-Strep beads and Western blotting to detect the polyubiquitination chain of NSP8. (**D and H**) The plasmids described (TRIM22 0, 0.25, 0.5, 1, 1.5, and 2 mg) were transfected into HEK-293T cells, followed by Western blotting with the indicated antibodies. (**E and G**) The plasmids described were transfected into HEK-293T cells, treated with MG132 for 8 h, and then detected by Western blotting with the indicated antibodies. Results expressed as mean + SD (*n* = 3 independent experiments). * indicates *P* < 0.05, ** indicates *P* < 0.01, and *** indicates *P* < 0.001, and statistics used Student’s *t*-test.

The sites on NSP8 that undergo ubiquitinations are lysine residues at 72 and 97 positions (Fig. 3A) (29). Therefore, we constructed NSP8-2K-R expression plasmids with these lysine residues mutated to arginine. After NSP8-2K-R overexpression, chase assay results showed that the half-life of NSP8 was significantly prolonged (Fig. S3A). Next, we constructed two mutant NSP8 plasmids: NSP8-K72R and NSP8-K97R. After expressing NSP8-K72R and NSP8-K97R in HEK-293T cells, the half-life of NSP8-K72R was found to be almost same as that of wild-type (WT) NSP8, whereas that of NSP8-K97R was significantly short (Fig. 3B; Fig. S3B). Moreover, TRIM22 did not promote the ubiquitination and degradation of NSP8-K97R (Fig. 3C and D). These results suggest that the K97 site of NSP8 may be a key modification site for its ubiquitination-mediated degradation. In contrast to that of NSP8-WT, MG132 treatment did not affect the NSP8-K97R level (Fig. 3E). Similarly, after MG132 treatment, NSP8-K0 level and its ubiquitination were not affected by TRIM22, which is consistent with the above results (Fig. S3C through E). To further demonstrate that K97 is a key ubiquitination site for NSP8, we first mutated the amino acid at position 97 of NSP8-K0 to lysine (NSP8-R97K). The results showed that the level of ubiquitination and degradation rate of NSP8-R97K were promoted by TRIM22 (Fig. 3F through H). These results suggest that TRIM22 significantly enhances ubiquitination of NSP8 at its K97 site, which triggers the proteasomal degradation of NSP8.

### TRIM22 inhibits replication of SARS-CoV-2

**Fig 4:**
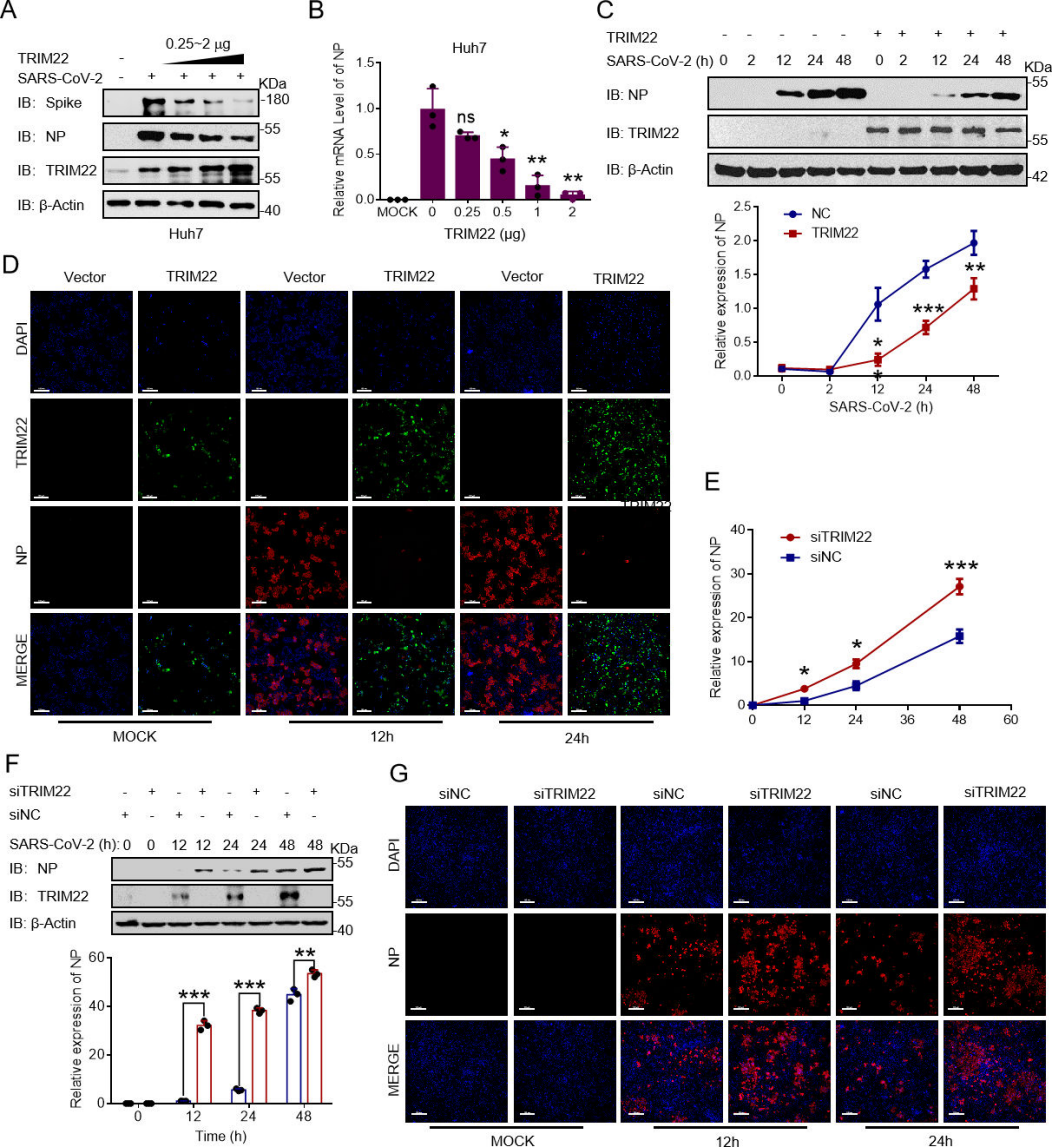
TRIM22 significantly inhibits the replication of SARS-CoV-2. (**A**) TRIM22 plasmids of 0, 0.25, 0.5, 1, and 2 µg were transfected into Huh7 cells and infected with the original SARS-CoV-2 virus for 24 h, and the indicated antibodies were used to detect Spike and NP protein. (**B**) TRIM22 plasmids of 0, 0.25, 0.5, 1, and 2 µg were transfected into Huh7 cells and infected with the original SARS-CoV-2 virus for 24 h. The NP RNA level of SARS-CoV-2 was detected by real-time quantitative PCR (RT-qPCR). (**C**) Vector or TRIM22 plasmids of 0, 0.25, 0.5, 1, and 2 µg were transfected into Huh7 cells and infected with the original SARS-CoV-2 virus for 0, 2, 12, 24, and 48 h, and the indicated antibodies were used to detect NP protein. (**D**) Vector or TRIM22 plasmids were transfected into hACE-Hela cells and infected with primary SARS-CoV-2 virus for 0, 12, and 24 h. Cells were analyzed by fluorescence microscopy. Strep-tagged NSP8 was labeled with anti-Strep antibody (red) and TRIM22 with anti-TRIM22 antibody (green). Cell nuclei were stained with 4',6-diamidino-2-phenylindole (DAPI) (blue). Representative images are shown. Scale bar, 100 µm. (**E**) The siNC or siTRIM22 plasmids were transfected into Huh7 cells and infected with the original SARS-CoV-2 virus for 0, 12, 24, and 48 h. The NP of SARS-CoV-2 mRNA level were detected by RT-qPCR. (**F**) The siNC or siTRIM22 plasmids were transfected into Huh7 cells and infected with the original SARS-CoV-2 virus for 0, 12, 24, and 48 h, and the indicated antibodies were used to detect NP and TRIM22 protein. (**G**) The siNC or siTRIM22 plasmids were transfected into Huh7 cells and infected with the original SARS-CoV-2 virus for 0, 12, and 24 h. Cells were analyzed by fluorescence microscopy. Strep-tagged NSP8 was labeled with anti-Strep antibody (red) and TRIM22 with anti-TRIM22 antibody (green). Cell nuclei were stained with DAPI (blue). Representative images are shown. Scale bar, 100 µm. Results expressed as mean + SD (*n* = 3 independent experiments). * indicates *P* < 0.05, ** indicates *P* < 0.01, and *** indicates *P* < 0.001, and statistics used Student’s *t*-test.

Because TRIM22 can target NSP8 for ubiquitination and degradation, we examined the effect of TRIM22 on SARS-CoV-2 replication. After gradient overexpression of TRIM22 in three different cell lines followed by SARS-CoV-2 infection, the levels of nucleocapsid and spike were examined. The results showed that TRIM22 overexpression markedly decreased the protein and RNA levels of SARS-CoV-2 (Fig. 4A and B; Fig. S4A through D). Meanwhile, viral load was significantly suppressed within 24 h post-infection, while beyond 24 h, viral load increased at a rate similar to that of the control (Fig. 4C). Immunofluorescence analysis showed that SARS-CoV-2 replication was significantly reduced after TRIM22 overexpression (Fig. 4D). We collected viruses from supernatants of infected cells expressing empty vector or TRIM22 and performed a cell infection assay. The results showed that the infection efficiency of an equal volume of supernatant containing daughter viruses was significantly reduced after TRIM22 overexpression (Fig. S4F and G). Small interfering RNA-mediated TRIM22 knockdown resulted in the upregulation of viral protein and RNA levels (Fig. 4E through G). This suggests that TRIM22 plays a vital role in SARS-CoV-2 replication.

### IFN-α stimulates TRIM22 expression to promote NSP8 degradation

**Fig 5:**
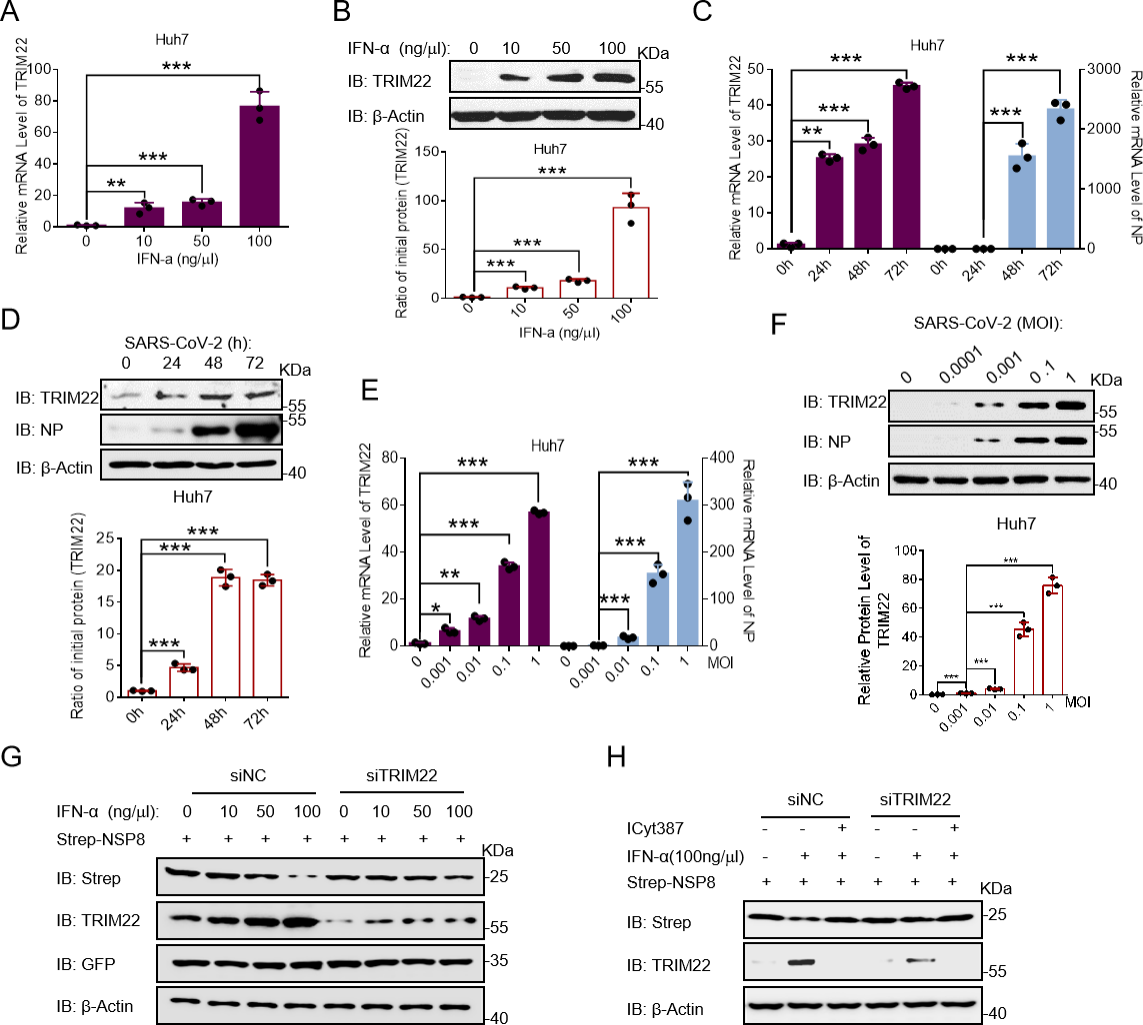
SARS-CoV-2 infection significantly stimulates high expression of TRIM22. (**A**) HEK-293T cells were treated with 0, 10, 50, and 100 ng/μL of IFN-a, and the expression of TRIM22 gene was detected by real-time quantitative PCR (RT-qPCR). (**B**) HEK-293T cells were treated with 0, 10, 50, and 100 ng/μL of IFN-a, and the indicated antibodies were used to detect TRIM22 protein. (**C**) Calu3 cells were infected with the original SARS-CoV-2 virus for 0, 24, 48, and 72 h, respectively, and the TRIM22 gene mRNA level was detected by RT-qPCR, and the extent of infection was detected by the NP gene of SARS-CoV-2. (D) Calu3 cells were infected with the original SARS-CoV-2 virus for 0, 24, 48, and 72 h, respectively, and the indicated antibodies were used to detect TRIM22 protein. (E) Calu3 cells were infected with the original SARS-CoV-2 virus at different MOI (0, 0.001, 0.01, 0.1, 1) for 24 h. The TRIM22 gene expression was detected by RT-qPCR and the degree of infection was detected by the NP gene of SARS-CoV-2. (F) Calu3 cells were infected with the original SARS-CoV-2 virus at different MOIs (0, 0.001, 0.01, 0.1, 1) for 24 h, and the indicated antibodies were used to detect TRIM22 protein. (G) Strep-NSP8 plasmid and IFN-α were added to Huh7 cells, and the corresponding proteins were detected with ant-Strep and anti-TRIM22 antibodies. (H) Strep-NSP8 plasmid, ICyt387 and IFN-α were added to Huh7 cells, and the corresponding proteins were detected with ant-Strep and anti-TRIM22 antibodies. Results expressed as mean + SD (*n* = 3 independent experiments). * indicates *P* < 0.05, ** indicates *P* < 0.01, and *** indicates *P* < 0.001, and statistics used Student’s *t*-test.

As an IFN-stimulated gene, TRIM22 is significantly upregulated by IFN stimulation (30). Therefore, we first verified whether TRIM22 expression was stimulated by IFN in Huh7 cells. The results showed that both the mRNA and protein levels of TRIM22 increased after IFN-α stimulation (Fig. 5A and B). The IFN signaling pathway is activated by SARS-CoV-2 (31). Therefore, we investigated whether TRIM22 expression was highly induced by SARS-CoV-2 using various cell lines (Calu3, Huh7, HEK 293T-hACE2, and HeLa-hACE2). The results showed that SARS-CoV-2 induced high expression of TRIM22 RNA (Fig. 5C; Fig. S4A through C). Furthermore, TRIM22 levels significantly increased with increasing duration of SARS-CoV-2 infection (Fig. 5D; Fig. S5D through F). In addition, the RNA and protein levels of TRIM22 gradually increased with increasing multiplicity of infection (MOI) of SARS-CoV-2 in different cell lines (Fig. 5E and F; Fig. S5G through L). Next, we examined the effects of IFN-α on NSP8 expression. The results showed that IFN-α treatment decreased NSP8 level, which was stabilized by TRIM22 knockdown (Fig. 5G). This effect disappeared with the addition of an inhibitor of the IFN signaling pathway (Fig. 5H). These results suggest that TRIM22 is highly expressed as an IFN-stimulated gene during SARS-CoV-2 infection and then degrades NSP8.

### Species-specific ubiquitination of NSP8 mediated by TRIM22

Because SARS-CoV-2 frequently mutates to escape host immunity (32–34), we compared the amino acid sequences of NSP8 from various SARS-CoV-2 strains and found that these sequences were identical (Fig. S6A and B; Table S2). This suggests that TRIM22 may inhibit the replication of various SARS-CoV-2 strains.

**Fig 6:**
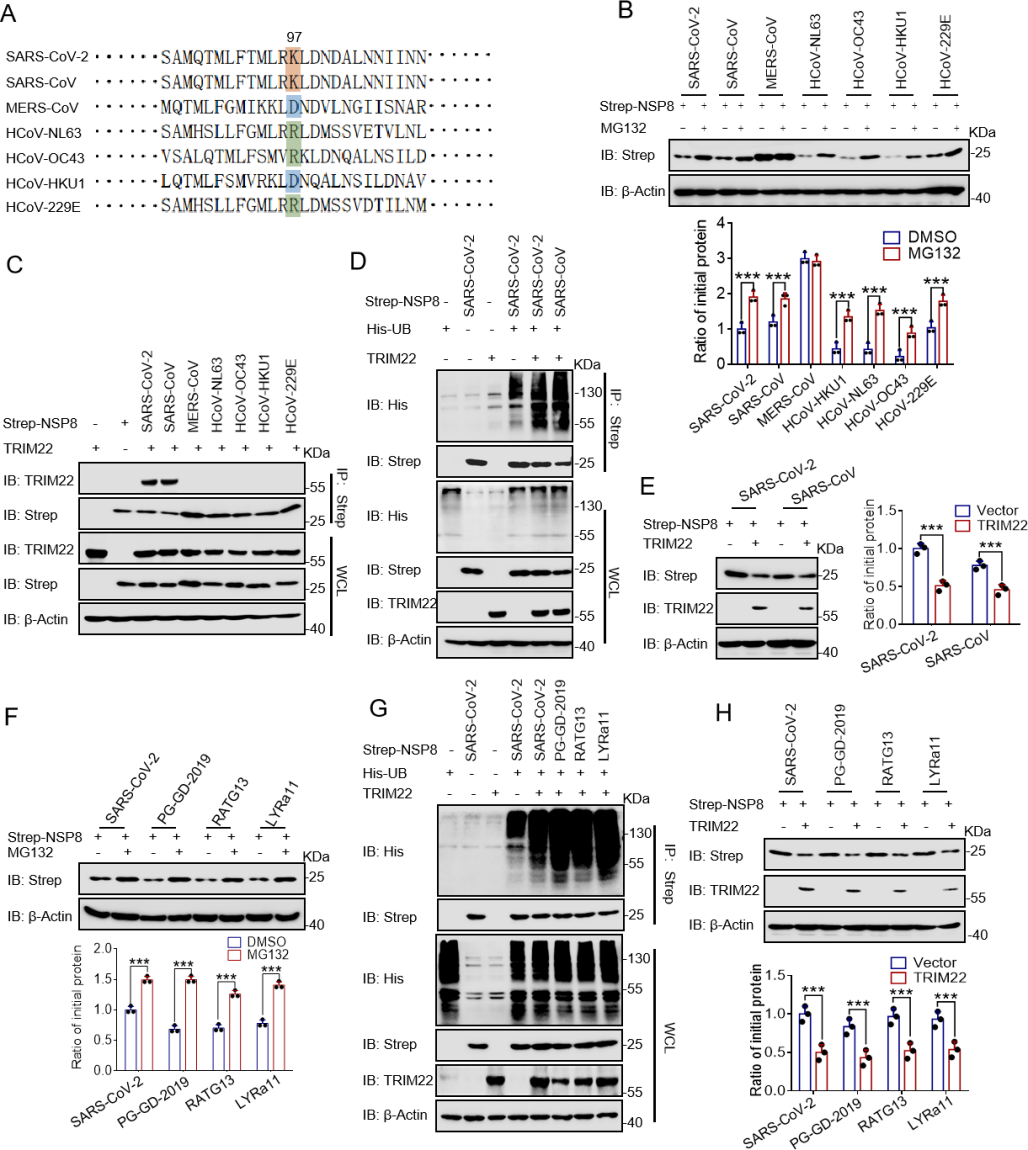
The degradation of NSP8 protein by ubiquitination by TRIM22 has a broad spectrum. (**A**) Schematic diagram of amino acid sequence alignment at the K97 ubiquitination site on NSP8 protein of coronavirus. (**B**) The described plasmids were transfected into HEK-293T cells and treated with MG132 (20 µM) for 4 h. Western blotting was performed with anti-Strep antibody. (**C**) The plasmids described were transfected into HEK-293T cells, and lysed with NP-40. The whole-cell lysates were subjected to pulldown with anti-Strep beads and Western blotting to detect TRIM22. (**D**) The plasmids described were transfected into HEK-293T cells and the ubiquitination of NSP8. Cells were treated with MG132 (20 µM) for 8 h prior to collection. The whole-cell lysates were subjected to pulldown with anti-Strep beads and Western blotting to detect the polyubiquitination chain of NSP8. (**E**) The plasmids described were transfected into HEK-293T cells, and then detected by Western blotting with the anti-Strep antibodies. Results expressed as mean + SD (*n* = 3 independent experiments). * indicates *P* < 0.05, ** indicates *P* < 0.01, and *** indicates *P* < 0.001, and statistics were passed Student’s *t*-test.

Next, we considered all the seven human coronaviruses, all of which expresses NSP8 (35). To determine whether TRIM22 inhibits the replication of other coronaviruses by promoting NSP8 degradation, we compared the amino acid sequences of NSP8 from all human coronaviruses. We found that the NSP8 sequence of SARS-CoV had a high similarity with that of SARS-CoV-2 (Fig. 6A; Fig. S6C; Table S2). We overexpressed plasmids containing NSP8 from all the human coronaviruses and treated them with MG132. The results showed that NSP8 levels, except for Middle East respiratory syndrome coronavirus (MERS-CoV), were significantly rescued by MG132 treatment (Fig. 6B). The results of CHX treatment also indicated that NSP8 of MERS-CoV only was relatively stable (Fig. S6D). Unexpectedly, co-IP results showed that NSP8 from SARS-CoV or SARS-CoV-2 only directly interacted with TRIM22 (Fig. 6C). TRIM22 promoted the ubiquitination of NSP8 from SARS-CoV (Fig. 6D). Moreover, NSP8 level of SARS-CoV was significantly downregulated after TRIM22 overexpression (Fig. 6E).

SARS-CoV and SARS-CoV-2 have been partially transformed from bat or pangolin coronaviruses (36–38). Therefore, we speculated that TRIM22 could promote the degradation of NSP8 from coronaviruses of other species. We compared amino acid sequences of NSP8 from pangolin (PG-DG-2019) and bat (RATG13 and LYRa11) coronaviruses. The results showed that NSP8 sequences of PG-DG-2019, RATG13, and LYRa11 were highly similar with that of SARS-CoV-2 (Fig. S6E and F; Table S2). NSP8 protein levels in all of them were rescued by MG132 treatment (Fig. 6F). The results of CHX treatment also indicated that NSP8 was relatively unstable in these species (Fig. S6G). Co-IP experiments showed that TRIM22 interacted with NSP8 of PG-DG-2019, RATG13, and LYRa11 and promoted ubiquitin-mediated degradation (Fig. 6H). These results suggest that TRIM22 inhibits viral replication by promoting ubiquitin-mediated degradation of NSP8 from a broad spectrum of coronaviruses. Therefore, drug development targeting this antiviral mechanism could be effective against various SARS-CoV-2 strains and coronaviruses of other species, which might transform into human coronaviruses in the future.

## DISCUSSION

Current efforts to combat SARS-CoV-2 transmission primarily focus on the stages of viral infection, such as vaccination to induce immunization (39–42) and antibodies targeting the spike protein (43, 44). However, owing to the high mutation rate of SARS-CoV-2, many immunization tools have become less effective (45–49). Given the highly conserved non-structural proteins, targeting them to develop new anti-SARS-CoV-2 therapies provides a natural advantage. NSP8 acts as a non-structural protein and directly binds to NSP7 and NSP12 to form RdRp (6, 7, 50, 51), facilitating SARS-CoV-2 replication. This suggests that NSP8 is a promising target for intervention. Liang et al. (25) have used a glutamate-terminal pattern-binding mechanism to confirm that TRIM7 can directly target NSP8 of SARS-CoV-2 and promote its ubiquitin-mediated degradation. Since the main objective of Liang et al. was not the stability of the RdRp complex, they neither systematically screened the E3 ligases of NSP8 nor examined the stability of other subunits of the RdRp complex. In our results, we systematically demonstrated that NSP8 is the only relatively unstable RdRp complex and can be directly induced for ubiquitin degradation by TRIM22.

The NSP8 protein is a non-structural protein critical for viral replication in SARS-CoV-2. Our results suggest that the degradation of NSP8 mediated by TRIM22, an interferon-stimulated gene, sheds light on the intricate mechanisms employed by the host immune system to combat viral infections. The identification of TRIM22 as a regulator in this process underscores the multifaceted nature of host-virus interactions, suggesting that host factors can exert control over viral protein stability and function. Firstly, we highlight the complexity of the host innate immune response against the virus. TRIM22 is an intrinsic component of the host defense mechanism and regulates viral replication by targeting specific viral protein degradation. Meanwhile, also for interferon treatment COVID-19 patients provides a new explanation. Moreover, the discovery of TRIM22-mediated degradation of NSP8 unveils a potential target for therapeutic intervention. Manipulating the ubiquitin-proteasome pathway to enhance the degradation of NSP8 could potentially hinder viral replication, offering a novel approach for drug development against COVID-19. Small molecules or compounds that mimic the action of TRIM22 could be explored to disrupt NSP8 stability and impede viral proliferation. These findings also prompt further investigations into the role of other host factors and their influence on viral protein stability. Understanding how various host proteins interact with different components of SARS-CoV-2 could unravel new targets for antiviral therapies or aid in the development of broad-spectrum antiviral strategies. Additionally, exploring the impact of TRIM22 on other viral proteins or its role in the context of viral mutants or variants could expand our understanding of its therapeutic potential and limitations. Furthermore, translating these findings into clinical applications warrants attention. Developing drugs or therapeutic agents that modulate TRIM22 activity or mimic its function could be a promising avenue.

**Fig 7:**
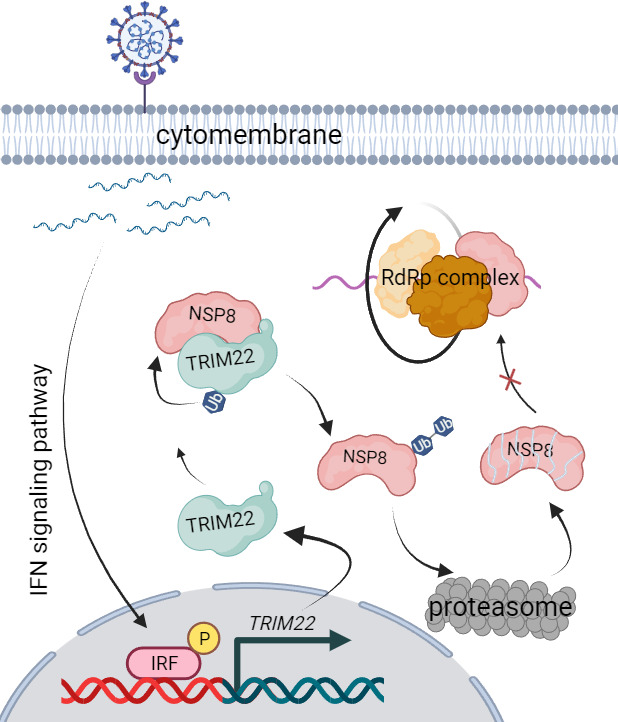
The abstract overview of this study. After SARS-CoV-2 invades host cells, the released viral molecules activate the IFN signaling pathway and promote TRIM22 expression. TRIM22 binds to the NSP8 protein of SARS-CoV-2 and promotes ubiquitin proteasomal degradation of NSP8, thereby inhibiting SARS-CoV-2 replication.

In conclusion, the discovery of TRIM22-mediated degradation of NSP8 in SARS-CoV-2 represents a significant advancement in our understanding of host-virus interactions (Fig. 7) . It opens doors for innovative therapeutic strategies and underscores the importance of exploring host factors as potential targets for combating viral infections like COVID-19. Further research in this direction holds promise for the development of novel antiviral interventions with the potential to impact global health positively.

## MATERIALS AND METHODS

### Cell lines

HeLa-hACE2, HEK-293T-hACE2, Huh7, and VeroE6 cells were cultured in Dulbecco’s modified Eagle medium (Gibco, 11965092) supplemented with 10% fetal bovine serum (04-001-1; Biological Industries, Beit-Haemek, Israel) and 100 µg/mL penicillin/streptomycin. Calu3 cells were cultured in minimal essential medium (Gibco, 11095080) supplemented with 20% fetal bovine serum and 100 µg/mL penicillin/streptomycin in a humidified 5% CO_2_ incubator at 37°C. All cell lines were permanently maintained in our laboratory and tested negative for mycoplasma. Among them, HeLa-hACE2 was constructed by expressing the hACE-receptor gene in HeLa cells in our laboratory.

### Immunoprecipitation and His pulldown

Cells were washed with phosphate buffered saline (PBS) and then lysed with 8 M urea lysis buffer (8 M urea, 100 mM NaH_2_PO_4_, 10 mM Tris-HCl pH 8.0, 500 mM NaCl, 10% glycerol, 0.1% Triton X-100, 10 mM β-ME, 10 mM imidazole, supplemented with protease inhibitor cocktail) for 1 h on ice. The lysate was incubated with Ni-NTA agarose (Invitrogen, 600442) and rotated for 4 h at room temperature. After incubation, agarose was washed with lysis buffer containing 20 mM imidazole. Proteins were eluted with elution buffer (0.15 M Tris-HCl pH 6.7, 5% SDS, 30% glycerol, 200 mM imidazole, and 0.72 M β-ME). Eluted samples were subjected to SDS-PAGE and Western blotting.

Cells were collected and lysed in lysis buffer (50 mM Tris at pH 7.5, 150 mM NaCl, 1% NP-40, and complete protease inhibitor cocktail tablets) at 4° for 1 h followed by centrifugation at 12,000 g for 10 min. Precleared cell lysates are mixed with antibody-coupled protein G agarose beads and incubated at 4°C overnight. The next day, the beads were washed at 4°C with wash buffer (20 mM Tris, pH 7.5, 100 mM NaCl, 0.1 mM EDTA, and 0.05% Tween 20) and centrifuged at 800 × *g* for 1 min. Proteins were eluted with elution buffer (0.1 M glycine-HCl, pH 2.5) and analyzed by SDS-PAGE and immunoblotting.

### Plasmids and antibodies

Flag-TRIM22 was obtained from SunV Biotechnology (Shenzhen, China), and the mutant plasmid for TRIM22 was obtained by site-directed mutagenesis on the original. Strep-NSP8 was kindly provided by Prof. Nevan J. Krogan (University of California, San Francisco), and the mutant plasmid for NSP8 was obtained by site-directed mutagenesis on the plasmid. His-Ub and His-UB-mutant plasmids were kindly provided by Prof. Junying Yuan (Center for Interdisciplinary Research in Biology and Chemistry, Shanghai Institute of Organic Chemistry, Chinese Academy of Sciences). si-TRIM7 and si-TRIM22 were purchased from Guangzhou Ribo Biotechnology Co.

Anti-His (TransGen Biotech, F1804), anti-TRIM22 (Proteintech, 13744-1AP), anti-TRIM7 (Proteintech, 26285-1-AP), anti-Strep tag II (Abcam, ab76949), anti-GFP (TransGen Biotech, HC101), anti-NSP8 (abclonal, A20202), anti-nucleocapsid protein (Sino Biological, 11675-T62), anti-Spike (Sino Biological, 40591-R235), anti-mouse IgG (TransGen Biotech HS201-01), anti-rabbit IgG (TransGen Biotech, HS101-01), and anti-actin (TransGen Biotech, HC201) were purchased from the companies indicated in parentheses.

### SARS-CoV-2 preparation and infection

All studies involving SARS-CoV-2 infection were conducted in the biosafety level laboratory at Shenzhen Third People’s Hospital. the SARS-CoV-2 isolate SZTH-003 was obtained from a patient with COVID-19. The genome sequence of SZTH-003 has been stored in the Global Influenza Data Sharing Initiative (Global Initiative on Sharing All Influenza Data [GISAID], EPI_ ISL_406594). SARS-CoV-2 was amplified once in VeroE6 cells, and the virus was stored at −80°C. The HeLa-hACE2, Huh7, VeroE6, and Calu3 cells were incubated with SARS-CoV-2 at different MOIs for 1 h at 37°C. Subsequently, the infected medium was removed, and the cells were cultured with normal medium at 37°C in an environment containing 5% CO_2_ and then harvested for analysis at the indicated time points.

### Real-time quantitative PCR (RT-qPCR)

RNA was isolated from cells using TRIzol reagent (15596-026; Invitrogen, Carlsbad, CA, USA). RNA was reverse transcribed using EasyScript First-Strand cDNA Synthesis SuperMix (AE301; TransGen Biotech, Beijing, China) for RNA reverse transcription. cDNA was stored at −80°C before use. RNA was isolated using Power SYBR Green PCR Master Mix (2×) (4367659; ABI, Carlsbad, CA, USA) on an Mx3005P instrument (Agilent Technologies, Stratagene, La. Technologies, Stratagene, La Jolla, CA, USA) for RT-qPCR). RT-qPCR amplification of target fragments was performed as follows: initial denaturation at 95°C for 2 min, followed by 45 cycles at 95°C for 15 s, 57°C for 15 s, and 68°C for 20 s. Data were normalized to the stewardship level. Data were normalized to the housekeeping β-actin gene and the relative abundance of target genes was calculated using the Ct model. The primers used RT-qPCR were human TRIM22-F: 5′-GAGAACCGCCTGGAAGATCG-3′; TRIM22-R: 5′-ACTGACGATCCCCTCAAC CT-3′; human GAPDH-F:5′-GGAGCGAGATCCCTCCAAAAT-3′; GAPDH-R: 5′-GGCTGTTGTCATACTTCTCATG-3′; SARS-CoV-2 (NP)-F: 5′-CGGAATGTCTC GCATCGGTA-3′; SARS-CoV-2(NP)-R: 5′-GGGCAACGCTTGTGTTTCAT-3′.

### Immunofluorescence

HeLa-hACE2 cells were cultured on coverslips and infected with SARS-CoV-2 for 24 h, then washed with PBS (Takara, T900) and fixed in 4% paraformaldehyde for 30 min at room temperature. After washing with PBS, cells were permeabilized with 0.5% Triton X-100 (Sangon Biotech, A110694) in PBS for 20 min at room temperature and then washed again with PBS. Cells were closed with 5% bovine serum albumin (BSA) (Beyotime, ST025) for 1 h and then incubated overnight at 4°C with the appropriate primary antibody. The next day, cells are incubated with fluorescently labeled secondary antibodies. Nuclei were stained with DAPI (Beyotime, C1102). After staining, stained cells are examined under a Leica DMi8 laser confocal microscope (Leica Microsystems).

### NSP8-host interactome

Ten trays of 10 cm dish HEK-293T cells were prepared and empty vector and NSP8 vector were transfected separately. Forty-eight hours later, cells were collected and treated with cell lysis buffer (50 mM Tris-HCl [pH 7. 4], 150 mM NaCl, 1% NP-40, 5 mM EDTA, 5% glycerol) supplemented with protease inhibitor cocktail (Bimake) treatment on ice for 1 h. Cell debris were removed by centrifugation at 12,000 rpm for 10 min at 4°C. Whole-cell lysates were transferred to a new tube and incubated with anti-Strep-II agarose beads at 4°C for 4 h, followed by centrifugation at 4,000 rpm for 5 min. Unbound proteins were washed three times with lysis buffer. Samples were eluted with protein loading buffer (0.01% bromophenol blue, 0.1 M dithiothreitol, 6% glycerol, 2% SDS, 0.25 M Tris-HCl [pH 6.8]) by boiling at 100°C for 5 min. After separation on SDS-PAGE gels, the samples were stained with Komas Brilliant Blue. The gels were recovered for mass spectrometry detection and analysis according to the location of NSP8 protein.

### *In vitro* ubiquitination

In the *in vitro* ubiquitination assay, Strep-NSP8 protein purified from *Escherichia coli* was incubated with UBE1 (100 ng), UBCH5a (150 ng), human recombinant Ub (5 mg, Boston Biochem; Cambridge, MA, USA) and human recombinant Ub (5 mg; Cambridge, MA, USA) in ubiquitination reaction buffer (Boston Biochem) for 90 min at 30°C. Co-IP incubation was performed with anti-Strep magnetic beads followed by Western blot analysis with anti-Ub antibody. The incubation mixture was co-IP using anti-Strep beads and then analyzed by protein blotting using anti-Ub antibody.

### Protein half-life assay

Polyethylenimine (PEI) transfection was performed when the cells in the 6 cm culture dish reached approximately 70% confluence. Transfection was performed when approximately 70% confluence was reached. Four milligrams of plasmid expressing NSP8 or the mutant was used for transfection. At 12 h post-transfection, cells were digested, resuspended, and evenly partitioned into wells in a 12-well plate. Twenty-four hours later, when cells reached approximately 90% confluence, they were treated with the protein synthesis inhibitor cytokinin. Cells were treated with the protein synthesis inhibitor CHX (30 mg/mL) or co-treated with MG132 (20 mM). Cells were then collected at the indicated times. The levels of target proteins were detected by Western blotting.

### Statistical analysis

The standard deviation of all results is presented as the mean of 6 SD. Student’s *t*-test (unpaired, two-tailed) was used to compare two independent groups, while two-way analysis of variance was performed for comparisons of multiple groups. All statistical analyses were performed using GraphPad Prism 7. *P* values of 0.05 were considered statistically significant. All experiments were repeated three times or more.

## Data Availability

The sequence numbers of the NSP8 reference genes are placed in Table S2 in the supplemental material, and all sequences can be searched in the Global Initiative of Sharing All Influenza Data (https://db.cngb.org/gisaid/).

## SUPPLEMENTAL MATERIAL

Supplemental figures mbio.02320-23-s0001.docx Fig. S1-6.

Supplemental tables mbio.02320-23-s0002.xlsx Tables S1 and S2.

ASM does not own the copyrights to Supplemental Material that may be linked to, or accessed through, an article. The authors have granted ASM a non-exclusive, world-wide license to publish the Supplemental Material files. Please contact the corresponding author directly for reuse.
