# Supplementary material for: The E3 ligase TRIM22 restricts SARS-CoV-2 replication by promoting proteasomal degradation of NSP8
Source: mBio. 2024 Jan 26;15(2):e02320-23. doi: 10.1128/mbio.02320-23 (PMC10865846; doi:10.1128/mbio.02320-23)
Supplement: Supplemental figures — Fig. S1-6. [file mbio.02320-23-s0001.docx]

Supplementary Materials for

The E3 ligase TRIM22 restricts SARS-CoV-2 replication by promoting proteasomal degradation of NSP8

mBio ID: mBio02320-23R1

Lujie Fan^1*^, Yuzheng Zhou^2*^, Xiafei Wei^2*^, Wei Feng^2^, Huimin Guo^2^, Yunfei Li^2^, Xiang Gao^2^, Jian Zhou^2^, Yezi Wu^2^, Xiaotong Shen^2^, Lei Liu^1,2#^, Gang Xu^3#^, Zheng Zhang^2, 4, 5#^

**Author affiliations and contact information**

^1^Guangzhou Laboratory, Guangzhou Medical University, Guangzhou, China.

^2^Institute for Hepatology, National Clinical Research Center for Infectious Disease, Shenzhen Third People's Hospital, The Second Affiliated Hospital, School of Medicine, Southern University of Science and Technology, 518112, Shenzhen, Guangdong Province, China.

^3^School of Basic Medical Sciences, Anhui Medical University, Hefei, China.

^4^Guangdong Key laboratory for anti-infection Drug Quality Evaluation, Shenzhen, Guangdong 518112, China.

^5^Shenzhen Research Center for Communicable Disease Diagnosis, Treatment of Chinese Academy of Medical Science, Shenzhen, Guangdong 518112, China.

Supplementary figures


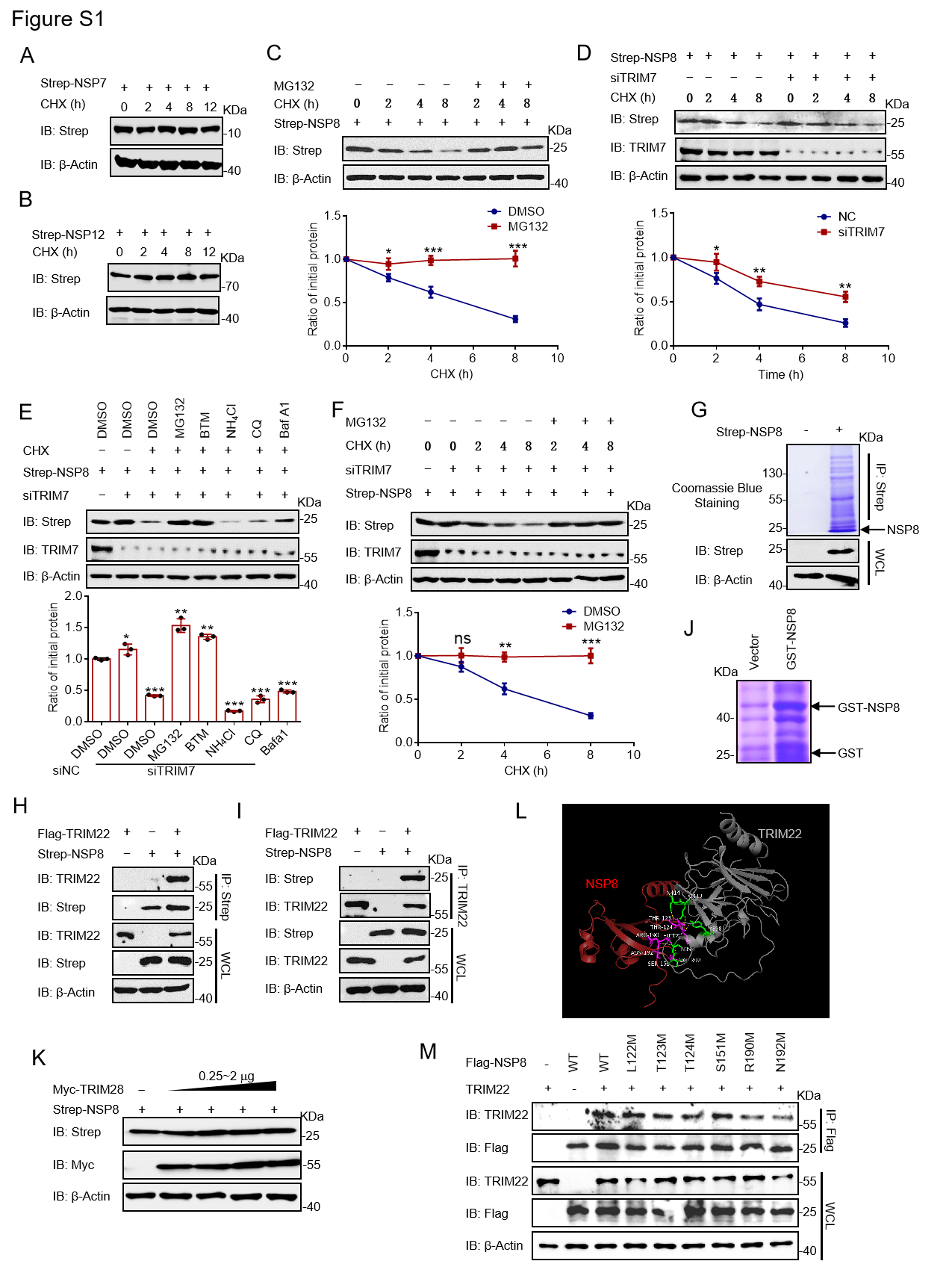


**Figure S1. NSP8 is degraded by the ubiquitin proteasome pathway and interacts with TRIM22.** (A, B) HEK-293T cells in a 6-cm dish were transfected with the indicated Strep-tagged plasmids. 12 hours later, cells were split evenly into wells of a 24-well plate and treated with CHX (25 μg/ml) when 100% confluence. Cells were collected at the indicated times and the levels of NSP7 and 12 were detected with anti-Strep antibody. (C) HEK293T cells transfected with the Strep-NSP8 expression plasmid were co-treated with CHX (30 μg/mL) and DMSO or with CHX (25 μg/ml) and MG132 (20 μM). Cells were collected at the indicated times and Western blotting was performed with anti-Strep antibody. (D) Cells were transfected with the described plasmids and collected at the indicated times, and Western blotting was performed with anti-Strep antibody and anti-TRIM7 antibody, quantitative display of NSP8 protein levels relative to β-actin. (E) HEK293T cells transfected with Strep-NSP8 and siRNA (siNC or siTRIM7) plasmids were treated with dimethyl sulfoxide (DMSO), CHX (25 μg/ml), MG132 (20 μM), bortezomib (BTM) (10 μM), chloroquine (CQ) (20 μM), and NH4Cl (10 mM) for 8 h. were then collected. Protein levels of NSP8 were detected by Western blotting. ORF4b protein levels are shown quantitatively relative to β-actin. (F) HEK293T cells transfected with the Strep-NSP8 expression plasmid were co-treated with CHX (30 μg/mL) and DMSO or with CHX (25 μg/ml) and MG132 (20 μM). Cells were collected at the indicated times and Western blotting was performed with anti-Strep antibody. (G) HEK293T cells transfected with Vector or Strep-NSP8, SDS-PAGE electrophoresis followed by Coomassie Blue Staining, presentation. (H, I) HEK-293T cells transfected with Flag-TRIM22 and Vector, Strep-NSP8 and Vector, and Flag-TRIM22 and Strep-NSP8 were lysed with NP-40. The cells were then divided into two parts, one for IP with anti-Strep antibody and the other for IP with anti-TRIM22 antibody. (J) The GST-NSP8 plasmid was introduced in *E. coli*, while the TRIM22 plasmid was expressed in HEK-293T cells, and the collected proteins were enriched and stained by SDS-PAGE electrophoresis and the target proteins were recovered. (K) Strep-NSP and Myc-TRIM28 (0, 0.25, 0.5, 1, 2μg)plasmids were transfected into HEK-293T cells, then detected by Western blotting with the indicated antibodies. (L) Protein interaction site prediction performed by <https://www.ebi.ac.uk/pdbe/pisa/.> (M) HEK-293T were transfected with the described plasmids, and then lysed with NP-40. The cells were then divided into two parts, one for IP with anti-Flag antibody and the other for whole cell lysates (WCL). Results expressed as mean + *SD* *(n = 3 independent experiments)*. * Indicates *P* < 0.05, ** indicates *P* < 0.01, and *** indicates *P* < 0.001, and statistics were passed *Student's* *t-test*.


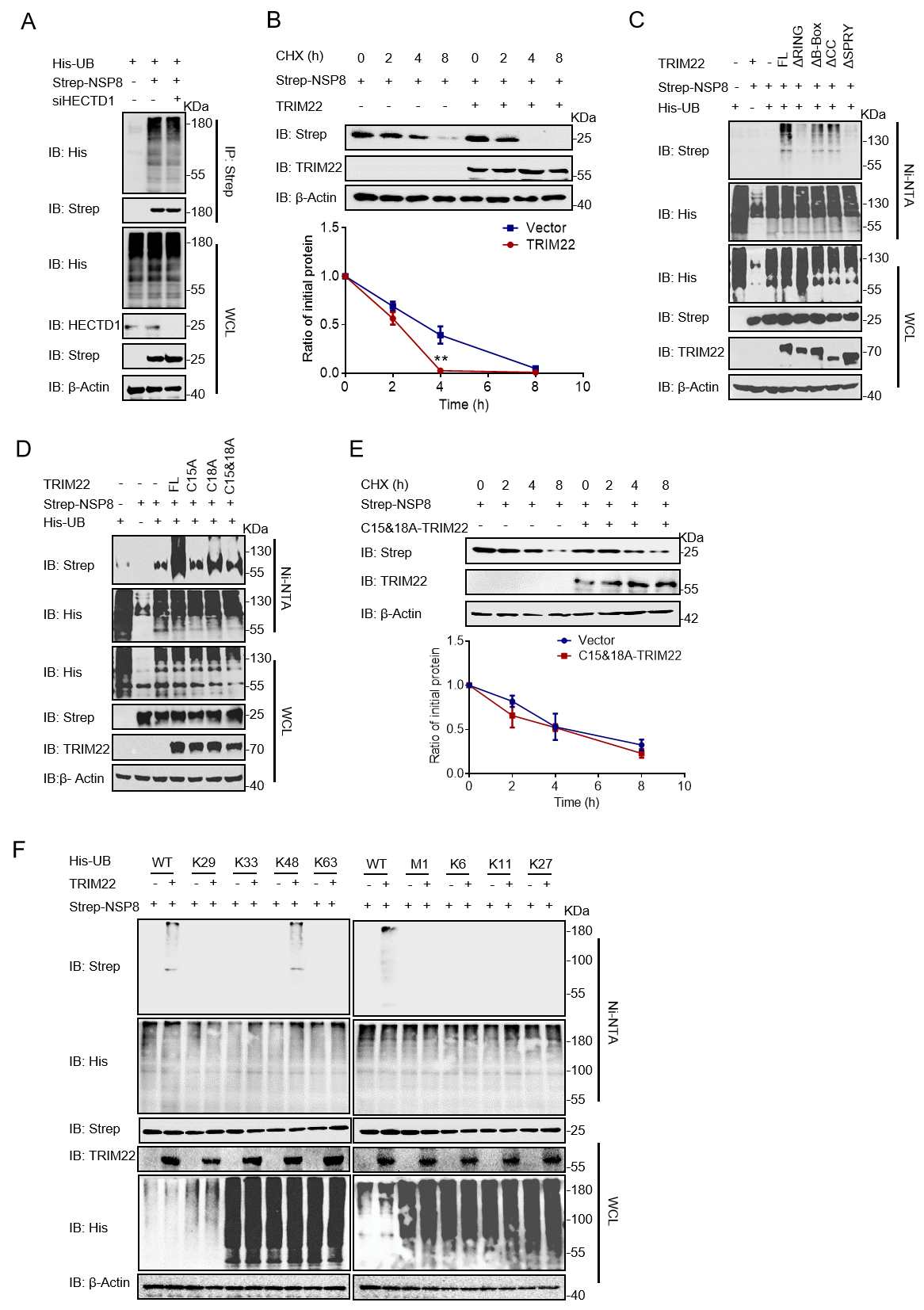


**Figure S2. TRIM22 promotes ubiquitin proteasomal degradation of NSP8.** (A) The NSP8 ubiquitination linkage was analyzed in HEK293T cells transfected with NSP8 and the indicated ubiquitin-WT, only retained Met-1, Lys-6, Lys-11, Lys-27 plasmids. Cells were treated with MG132 (20 μM) or not for 8 hours prior to collection. The whole-cell lysates were subjected to pulldown with Ni-NTA beads and Western blotting to detect the polyubiquitination chain of NSP8. (B, D) HEK-293T cells in a 6-cm dish were transfected with the indicated Strep-tagged plasmids. 12 hours later, cells were split evenly into wells of a 24-well plate and treated with CHX (25 μg/ml) when 100% confluence. Cells were collected at the indicated times and the levels of NSP7 and 12 were detected with anti-Strep antibody. (C) The described plasmids were transfected into HEK-293T cells and detected with Anti-TRIM22 antibody. (E, F) The NSP8 ubiquitination linkage was analyzed in HEK293T cells transfected with the described plasmids. Cells were treated with MG132 (20 μM) or not for 8 hours prior to collection. The whole-cell lysates were subjected to pulldown with Ni-NTA beads and Western blotting to detect the polyubiquitination chain of NSP8. Results expressed as mean + *SD* *(n = 3 independent experiments)*. * Indicates *P* < 0.05, ** indicates *P* < 0.01, and *** indicates *P* < 0.001, and statistics were passed *Student's* *t-test*.

**
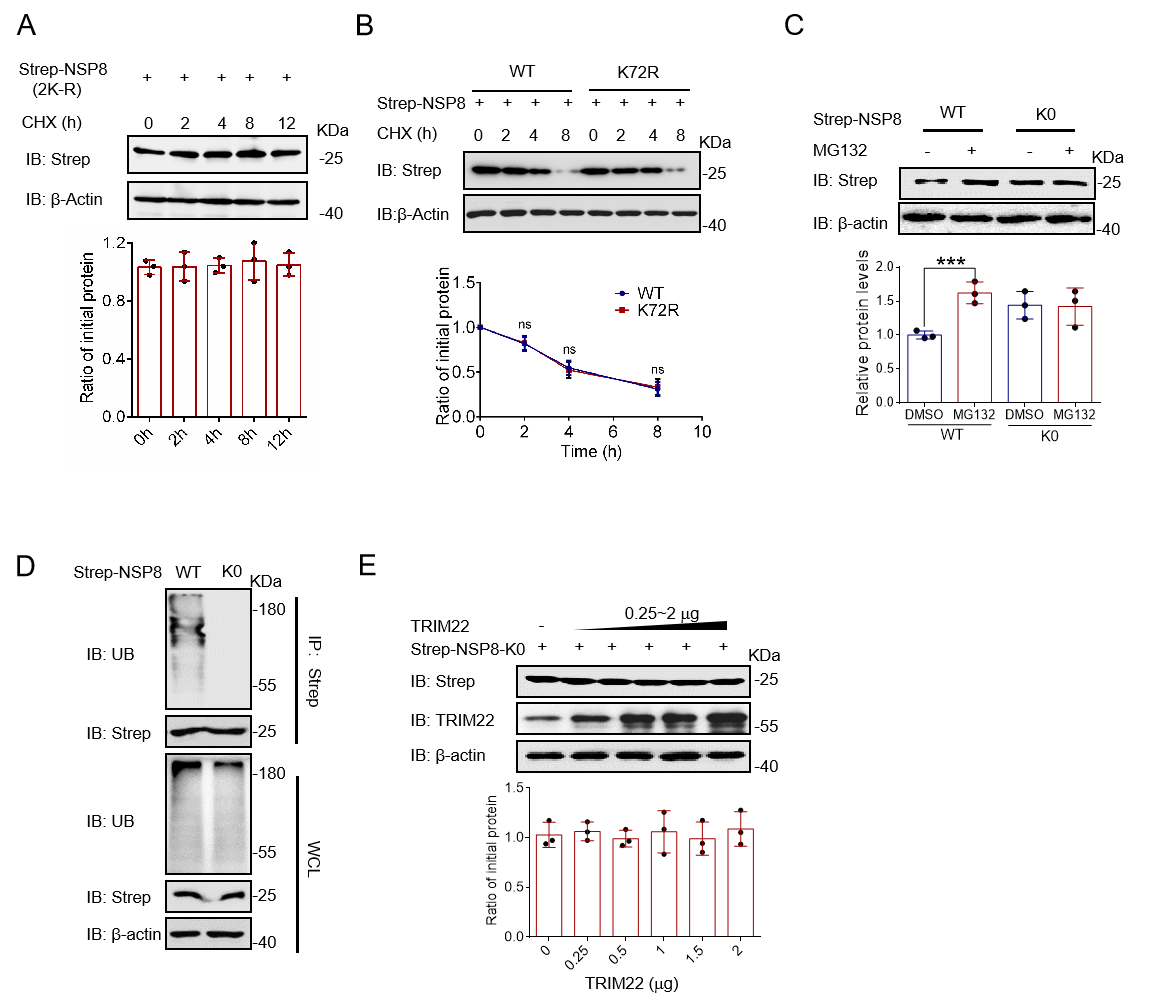
**

**Figure S3. TRIM22 induces the degradation of NSP8 by enhancing its ubiquitination on K97.** (A) HEK-293T cells in a 6-cm dish were transfected with the NSP8-K72&97R (2KR) plasmids. 12 hours later, cells were split evenly into wells of a 24-well plate and treated with CHX (25 μg/ml) when 100% confluence. Cells were collected at the indicated times and the levels of NSP8 were detected with anti-Strep antibody. (B) NSP8-WT or NSP8-K72R plasmids were transfected into HEK-293T cells, treated with CHX (25 μg/ml), and then detected by Western blotting with the indicated antibodies. (C) NSP8-WT or NSP8-K0 plasmids with Strep tags were transfected into HEK-293T cells, treated with MG132 for 8 h, and then detected by Western blotting with the indicated antibodies. (D) The plasmids described were transfected into HEK-293T cells and the ubiquitination of NSP8. Cells were treated with MG132 (20 μM) for 8 hours prior to collection. The whole-cell lysates were subjected to pulldown with anti-Strep beads and Western blotting to detect the polyubiquitination chain of NSP8. (E) The plasmids described plasmids (TRIM22 0, 0.25, 0.5, 1, 1.5 and 2 mg) were transfected into HEK-293T cells, followed by Western blotting with the indicated antibodies. Results expressed as mean + *SD* *(n = 3 independent experiments)*. * Indicates *P* < 0.05, ** indicates *P* < 0.01, and *** indicates *P* < 0.001, and statistics were passed *Student's* *t-test*.

**
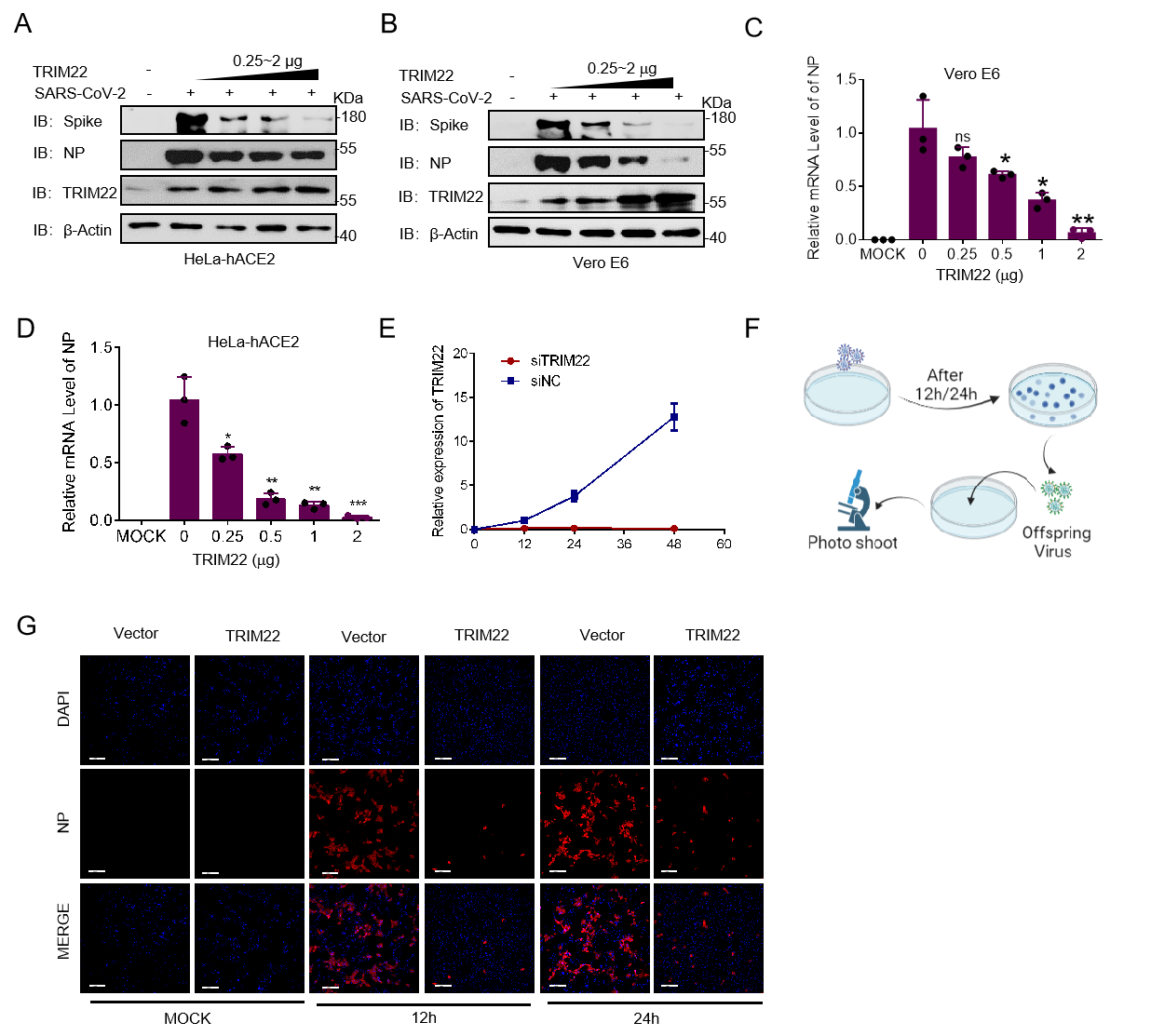
**

**Figure S4. TRIM22 significantly inhibits the replication of SARS-CoV-2.** (A, B) TRIM22 plasmids of 0, 0.25, 0.5, 1, and 2 μg were transfected into HeLa-hACE2 and Vero E6 cells and infected with the original SARS-CoV-2 virus for 24 h, and the indicated antibodies were used to detect Spike and NP protein. (C, D) TRIM22 plasmids of 0, 0.25, 0.5, 1 and 2 μg were transfected into HeLa-hACE2 cells and infected with the original SARS-CoV-2 virus for 24 h. The NP gene expression level of SARS-CoV-2 was detected by TR-qPCR. (E) The siNC or siTRIM22 plasmids were transfected into Huh7 cells and infected with the original SARS-CoV-2 virus for 0, 12, 24 and 48 h. The TRIM22 mRNA level were detected by RT-qPCR. (F) Schematic diagram of progeny virus infection. (G) Vero E6 cells were infected with progenitor viruses derived from exogenously expressed Vector or TRIM22, with red representing NP protein and blue representing nucleus. Results expressed as mean + *SD* *(n = 3 independent experiments)*. * Indicates *P* < 0.05, ** indicates *P* < 0.01, and *** indicates *P* < 0.001, and statistics were passed *Student's* *t-test*.

**
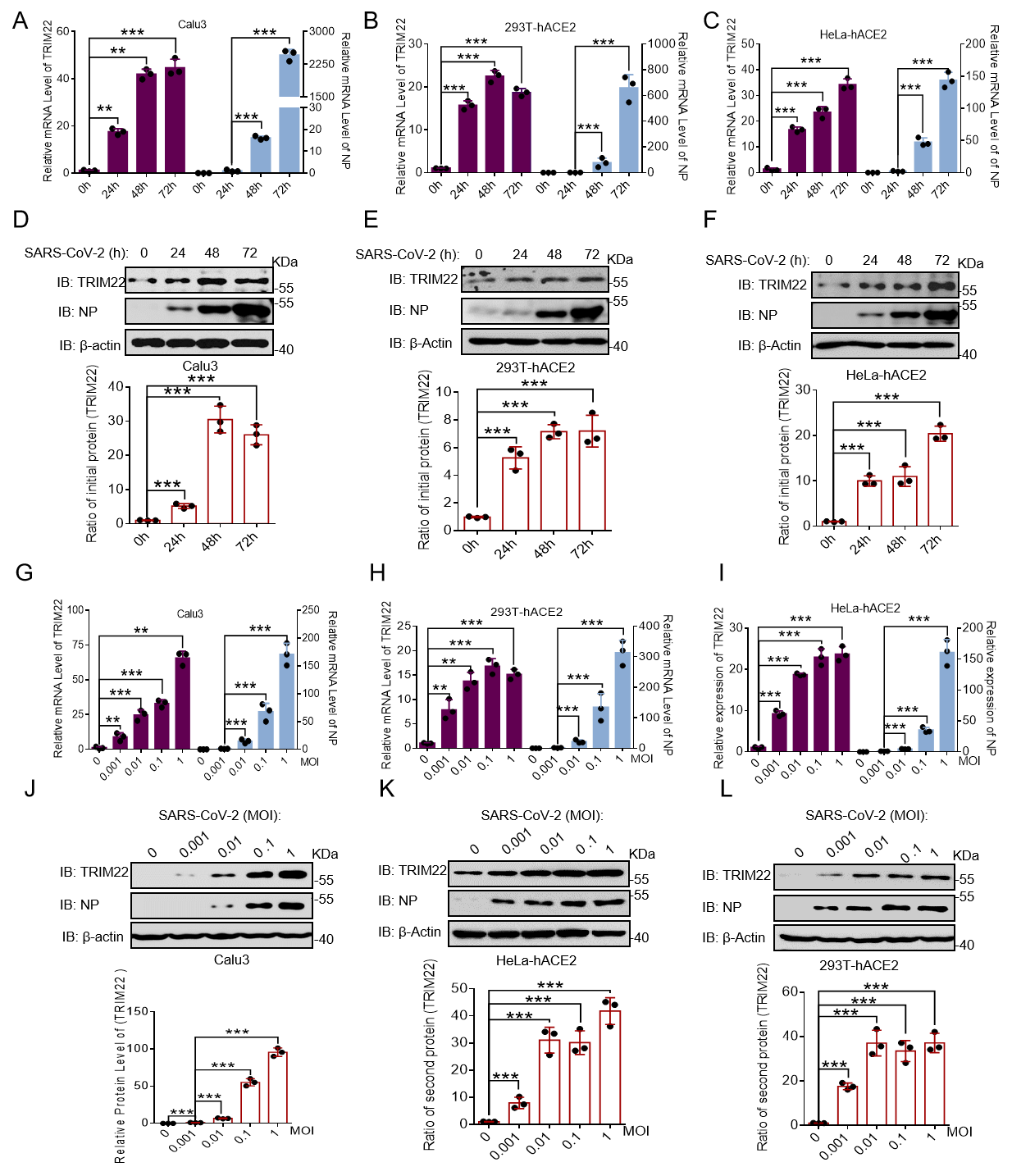
**

**Figure S5. SARS-CoV-2 infection significantly stimulates high expression of TRIM22.**  (A, B, C) Calu3, HEK-293T-hACE2 and HeLa-hACE2 cells were infected with the original SARS-CoV-2 virus for 0, 24, 48 and 72 hours, respectively, and the TRIM22 gene mRNA level was detected by RT-qPCR, and the extent of infection was detected by the NP gene of SARS-CoV-2. (D, E, F) Calu3, HEK-293T-hACE2 and HeLa-hACE2 cells were infected with the original SARS-CoV-2 virus for 0, 24, 48, and 72 h, respectively, and the indicated antibodies were used to detect TRIM22 protein. (G, H, I, J) Huh7, Calu3, HEK-293T-hACE2 and HeLa-hACE2 cells were infected with the original SARS-CoV-2 virus at different MOI (0, 0.001, 0.01, 0.1, 1) for 24 h. The TRIM22 gene expression was detected by RT-qPCR and the degree of infection was detected by the NP gene of SARS-CoV-2. (K, L, M, N) Huh7, Calu3, HEK-293T-hACE2 and HeLa-hACE2 cells were infected with the original SARS-CoV-2 virus at different MOI (0, 0.001, 0.01, 0.1, 1) for 24 h. Respectively, and the indicated antibodies were used to detect TRIM22 protein. Results expressed as mean + *SD* *(n = 3 independent experiments)*. * Indicates *P* < 0.05, ** indicates *P* < 0.01, and *** indicates *P* < 0.001, and statistics were passed *Student's* *t-test*.

**
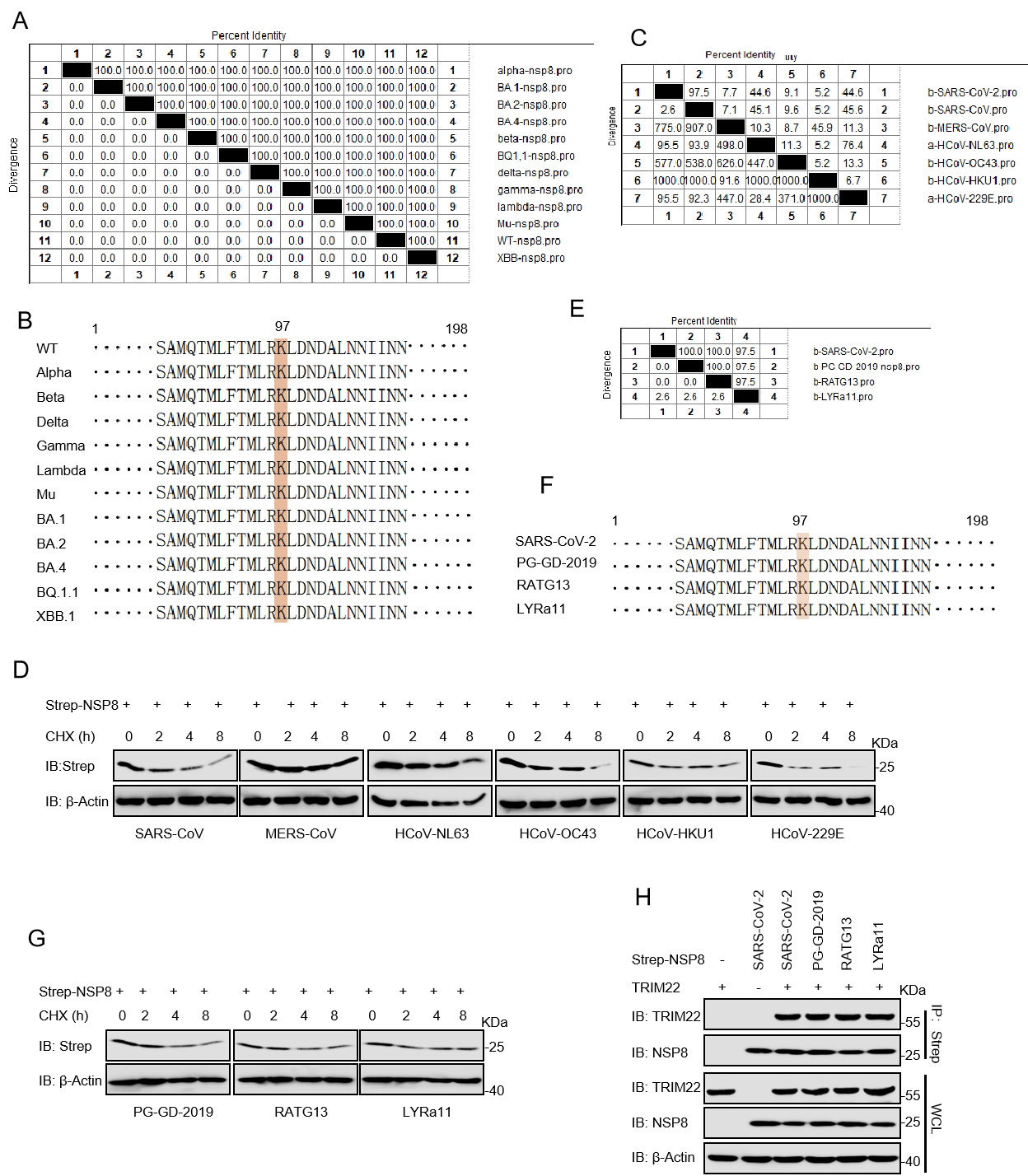
**

**Figure S6. The degradation of NSP8 protein by ubiquitination by TRIM22 has a broad spectrum.**  (A) Amino acid sequence similarity of NSP8 protein in major SARS-CoV-2 mutant. (B) Schematic diagram of amino acid sequence alignment at the K97 ubiquitination site on NSP8 protein in major SARS-CoV-2 mutant. (C) Amino acid sequence similarity of NSP8 protein in 7 hCoVs. (D) Amino acid sequence similarity of NSP8 protein in in some β-CoVs. (E) Schematic diagram of amino acid sequence alignment at the K97 ubiquitination site on NSP8 protein in in some β-CoVs. The described plasmids were transfected into HEK-293T cells and treated with MG132 (20 μM) for 4 h. Western blotting was performed with anti-strep antibody. (G) The plasmids described were transfected into HEK-293T cells, and lysed with NP-40. The whole-cell lysates were subjected to pulldown with anti-Strep beads and Western blotting to detect TRIM22. (H) The plasmids described were transfected into HEK-293T cells and the ubiquitination of NSP8. Cells were treated with MG132 (20 μM) for 8 hours prior to collection. The whole-cell lysates were subjected to pulldown with anti-Strep beads and Western blotting to detect the polyubiquitination chain of NSP8. (I) The plasmids described were transfected into HEK-293T cells, and then detected by Western blotting with the anti-Strep antibodies. Results expressed as mean + *SD* *(n = 3 independent experiments)*. * Indicates *P* < 0.05, ** indicates *P* < 0.01, and *** indicates *P* < 0.001, and statistics were passed *Student's* *t-test*.

**Supplementary Table legend**

**Table.1** The differences in protein abundance between Vector and NSP8 group identified by MS.

**Table. 2** Reference of NSP8 gene number in NCBI library.
